# The Role of Red Cell Distribution Width (RDW), RDW/Platelet Ratio, and Mean Platelet Volume as Prognostic Markers in Acute Pancreatitis Severity and Complications Based on the Bedside Index for Severity in Acute Pancreatitis Score

**DOI:** 10.7759/cureus.66193

**Published:** 2024-08-05

**Authors:** Amira Jagodić Ejubović, Malik Ejubović, Rijad Jahić, Amer Brkovic, Emina Letic, Milorad Vujnic, Orhan Lepara, Avdo Kurtović, Minela Becirovic, Emir Becirovic, Almir Fajkić

**Affiliations:** 1 Department of Internal Medicine, Cantonal Hospital Zenica, Zenica, BIH; 2 Department of Pathophysiology, University of Zenica, Zenica, BIH; 3 Department of Internal Medicine and Cardiology, University Clinical Center Sarajevo, Sarajevo, BIH; 4 Department of Surgery, General Hospital "Prim.dr. Abdulah Nakaš", Sarajevo, BIH; 5 Department of Pathophysiology, University of Banja Luka, Banja Luka, BIH; 6 Department of Human Physiology, Faculty of Medicine, University of Sarajevo, Sarajevo, BIH; 7 Department of Orthopedics and Traumatology, University Clinical Center Tuzla, Tuzla, BIH; 8 Department of Internal Medicine and Nephrology, University Clinical Center Tuzla, Tuzla, BIH; 9 Department of Internal Medicine, University Clinical Center Tuzla, Tuzla, BIH; 10 Department of Pathophysiology and Internal Medicine, Faculty of Medicine, University of Sarajevo, Sarajevo, BIH

**Keywords:** rdw/plt, rdw, inflammation, bisap score, pancreatitis

## Abstract

Background

Acute pancreatitis (AP) is a condition with various etiological factors, marked by the sudden onset of inflammation in the pancreatic tissue. Predicting the severity and potential mortality of AP involves analyzing clinical data alongside laboratory tests and imaging. Among several grading methods with strong predictive capabilities for illness severity and mortality, the Bedside Index for Severity in Acute Pancreatitis (BISAP) score is notable. This study aims to explore the potential role of laboratory markers, specifically red cell distribution width (RDW), RDW/platelet (PLT) ratio, and mean platelet volume (MPV), in predicting disease severity, with patients being stratified according to the BISAP scoring system.

Materials and methods

This research included 161 patients hospitalized at Cantonal Hospital Zenica in Zenica, Bosnia and Herzegovina, with a diagnosis of AP. The BISAP score was determined based on laboratory and radiological analyses. This score was used to evaluate potential correlations between laboratory findings such as RDW, RDW/PLT ratio, and MPV.

Results

The age range was significantly higher in patients with BISAP scores ≥3 (68 years, 64-76) compared to those with BISAP scores <3 (59.5 years, 42.75-69) (p = 0.000). RDW values were also significantly higher in patients with BISAP scores ≥3 (15.6%, 14-16.9) compared to those with BISAP scores <3 (13.5%, 13-14.1) (p = 0.000). Hospital stay duration was significantly longer for patients with BISAP scores ≥3 (9 days, 6-11) compared to those with BISAP scores <3 (5 days, 5-7) (p = 0.000). Additionally, the RDW/PLT ratio was significantly lower in patients with BISAP scores <3 (0.063 ± 0.02) compared to those with BISAP scores ≥3 (0.09 ± 0.059) (p = 0.012).

Conclusion

Our results indicate a significant difference in RDW/PLT ratios between patient severity groups based on BISAP scores (scores <3 vs. ≥3). This suggests that the RDW/PLT ratio may serve as a useful predictor for assessing the severity of AP. However, further research is needed to explore the full potential of the RDW/PLT ratio in evaluating AP patients.

## Introduction

Acute pancreatitis (AP) is a multifactorial disease characterized by the sudden onset of inflammation in the pancreatic tissue. It is a common cause of hospital admission among gastrointestinal diseases [1]. The severity of AP ranges from mild, self-limiting cases to severe forms with significant complications, potentially leading to multiorgan failure and death [2]. Approximately 80% of patients experience a mild edematous form of AP, while the remaining 20% face severe or complicated courses, which can result in early or delayed systemic and local complications [3].

Abdominal pain, typically severe and persistent, often located in the epigastric region and radiating to the back, along with elevated serum lipase levels (at least three times the upper limit of normal), are the primary clinical indicators used to diagnose AP [4]. Given that 10-20% of patients with severe AP may not survive, it is crucial to identify those at risk of developing severe disease as early as possible [5]. There is significant interest in developing rapid and cost-effective biomarkers for accurate prognosis prediction at the time of hospital admission.

To predict the severity and potential mortality of AP, clinical data, laboratory tests, and imaging techniques are utilized. Several severity rating systems are employed to evaluate the disease [4]. Among these, the Bedside Index for Severity in Acute Pancreatitis (BISAP) has demonstrated strong predictive power for both illness severity and mortality [6]. In addition to various scoring systems, laboratory findings play a crucial role in assessing the severity of AP as well as its complications and potential mortality risk. Research has indicated that red cell distribution width (RDW) is a prognostic marker with a predictive value comparable to the BISAP score, particularly in forecasting the development of persistent organ failure [1].

This study aimed to explore the potential role of various laboratory findings, including RDW, RDW/platelet (PLT) ratio, and mean platelet volume (MPV), as prognostic markers for disease severity in AP. Specifically, we investigated whether these markers could be utilized to assess disease severity in patients stratified by the BISAP scoring system. Our primary hypothesis was that RDW, RDW/PLT ratio, and MPV are significant prognostic indicators of disease severity when stratified according to the BISAP score. To our knowledge, no similar studies have been conducted in our country.

## Materials and methods

This study included adult patients diagnosed with AP who were hospitalized at the Department of Internal Medicine with Hemodialysis at Cantonal Hospital Zenica, in Zenica, Bosnia and Herzegovina, between January 2022 and December 2023.

According to the 2012 update of the Atlanta classification, AP is diagnosed if at least two of the following three criteria are met: abdominal discomfort typical of AP, characterized by severe, ongoing pain that often radiates to the back; serum lipase (or amylase) activity at least three times higher than the upper limit of normal; and imaging features of AP observed through contrast-enhanced computed tomography, trans-abdominal ultrasound, or less frequently, magnetic resonance imaging [7].

The BISAP score was utilized to categorize patients early in their AP episode based on their likelihood of severe illness or mortality. Patients were divided into two groups: those with mild to moderately severe AP (BISAP score <3) and those with severe pancreatitis (BISAP score ≥3). Evidence suggests that a BISAP score of ≥3 is a reliable indicator for identifying high-risk AP patients [8].

Only the initial admission was used for enrolling patients with recurrent pancreatitis. The study excluded individuals under 18, those with metastatic tumors, AIDS, uremia, advanced liver cirrhosis, treatment-resistant heart failure, pancreatic cancer or chronic pancreatitis, those on immunosuppressive medications, and confirmed pregnancies.

At the time of admission, demographic data for each enrolled patient were collected and documented. Laboratory and clinical parameters were also measured and recorded, including white blood cell count, hemoglobin level, PLT level, RDW, MPV, hematocrit, renal and hepatic function, and electrolyte levels. The BISAP score was assessed upon admission according to international criteria and evaluated using the least favorable parameters available within the first 24 hours. Patients with AP were monitored throughout their hospital stay, and data on intra-hospital mortality were collected. Based on their prognosis, patients were categorized into survival and death groups.

Data analysis

Statistical analysis was conducted using MS Excel (Microsoft Office Excel 2010; Microsoft Corporation, Redmond, WA, USA) and IBM SPSS Statistics for Windows, Version 22.0 (IBM Corp., Armonk, NY, USA). The Shapiro-Wilk test was employed to assess the normality of variable distributions. For continuous independent variables with a normal distribution, means and standard deviations were calculated, while medians and IQRs were used for those without a normal distribution. Student’s t-test was applied to determine the significance of differences for normally distributed variables, whereas the Mann-Whitney U-test was used for variables that did not follow a normal distribution. A p-value of <0.05 was considered statistically significant.

## Results

Table 1 presents the basic demographic and laboratory data of patients. Age was significantly higher in patients with a BISAP score ≥3, with a median of 68 (64-76), compared to those with a BISAP score <3, who had a median age of 59.5 (42.75-69) (p = 0.000). Additionally, RDW was significantly higher in patients with a BISAP score ≥3, with a median of 15.6 (14-16.9), compared to those with a BISAP score <3, who had a median RDW of 13.5 (13-14.1) (p = 0.000).

**Table 1:** Comparison of demographic and laboratory values between patients with AP categorized by BISAP scores <3 and ≥3 Data are expressed as mean (± SD) and median (IQR). * p < 0.05; ** p < 0.01 AP, acute pancreatitis; BISAP, Bedside Index for Severity in Acute Pancreatitis Score; MPV, mean platelet volume; PLT, platelet; RDW, red blood cell distribution width

Parameter	BISAP <3 (130)	BISAP ≥3 (31)	p
Male/female	69/61	10/22	0.000**
Age (years)	59.5 (42.75-69)	68 (64-76)	0.000**
MPV	9.5 (8.4-10.4)	9.3 (8-10.7)	0.712
RDW	13.5 (13-14.1)	15.6 (14-16.9)	0.000**
Thrombocyte (10^9^)	231 (191.75-279.25)	220 (168-277)	0.285
RDW/thrombocytes	0.063 ± 0.02	0.09 ± 0.059	0.012*
Days in the hospital	5 (5-7)	9 (6-11)	0

The hospital stay was statistically significantly longer for patients with a BISAP score ≥3, averaging 9 (6-11) days, compared to 5 (5-7) days for those with a BISAP score <3 (p = 0.000). Additionally, the RDW/PLT ratio was significantly lower in patients with BISAP <3, at 0.063 ± 0.02, compared to 0.09 ± 0.059 in patients with BISAP ≥3 (p = 0.012) (Table 2).

**Table 2:** Demographic and laboratory values of patients with AP Data are expressed as mean (±SD) and median (IQR). AP, acute pancreatitis; MPV, mean platelet volume; PLT, platelet; RDW, red blood cell distribution width

Parameter	Values
Male/female	79/82
Age (years)	62 (45-71)
MPV	9.4 (8.4-10.4)
RDW	13.7 (13.02-14.77)
PLT (10^9^)	230 (184-278)
RDW/PLT	0.07 ± 0.03
Hospital stay	6 (5-8)

## Discussion

This study aimed to explore the correlation between AP severity and various complete blood count-derived tests, including RDW, MPV, and the RDW/PLT ratio. Laboratory findings guide clinicians toward critical clinical decisions, offering more predictive value when combined with established assessment scales such as the BISAP score. Despite a lower incidence of AP from 1990 to 2019, mortality rates remained high and varied between countries. Notably, alcohol-related causes contributed significantly to AP-related deaths, particularly in highly developed and upper-middle-income regions [9].

The clinical diagnosis of AP involves meeting two or more specific criteria, with chronic therapy or previous viral infection playing a significant role in the diagnostic process [10]. While biochemical parameters may correlate with the severity of AP, recent research has highlighted the utility of new scoring systems such as the APACHE II score, BISAP score, and Ranson criteria. Notably, Gao et al. found that the BISAP score demonstrated higher sensitivity and specificity compared to both the Ranson criteria and the APACHE II score [8].

Our study found a statistically significant difference between patient groups with BISAP scores of less than 3 and those with scores of 3 or greater, specifically regarding the RDW/PLT ratio and RDW values. RDW values were significantly correlated with the duration of the hospital stay. Consistent with our findings, Karabuga et al. reported a significant correlation between RDW values and AP severity as assessed by the BISAP score [1]. Furthermore, Rezan et al. indicated that RDW values are significantly associated with the BISAP score, aiding in the prediction of AP mortality and severity [11].

Compared to other AP scoring systems, which showed slight inferiority in assessing severity outcomes, the RDW value at admission correlated with the 48-hour Ranson score, as suggested by Kılıç et al. [12]. This underscores the potential of RDW to provide valuable information regarding AP severity at the time of admission. Finally, Cheng et al. published a systematic review with meta-analysis that used RDW as a predictor of severity in AP patients. Their findings suggested that changes in RDW values were significantly positively correlated with disease severity [13].

When comparing our results on hospital stay and RDW values with previous research, we found that RDW was positively correlated with longer hospital stays, consistent with earlier findings [14,15]. Çetinkaya et al. proposed that the RDW/PLT ratio could be a predictor of mortality in AP patients, highlighting it as one of the initial studies on this topic. However, this research did not compare the RDW/PLT ratio to established AP scoring systems [16]. Currently, there are no published studies that link the RDW/PLT ratio to the AP severity scale.

The potential relationship between the mentioned blood-derived tests and AP pathophysiology may primarily involve PLT and their interaction with PLT-activating factor (PAF). During AP, PAF levels are elevated and are associated with multi-organ failure in various conditions. PAF is primarily produced by leukocytes. Additionally, inflammatory cytokines such as tumor necrosis factor alpha and IL-6 are increased in this context [17].

Current literature indicates that PAF activity is indirectly related to PAF acetylhydrolase (PAFAH) levels. Research has shown that PLT counts are typically higher in patients with elevated PAFAH levels, suggesting an inverse relationship between PLT count and PAFAH levels. This association was observed in patients with septic shock but did not show a significant correlation in the study by Tsukioka et al. involving AP patients [18,19].

Consequently, the RDW/PLT ratio’s dividend has led to research into RDW as an inflammatory marker in various pathological conditions, including hepatitis B, skin burns, and sepsis. In these studies, RDW values consistently correlated positively with disease severity and mortality, indicating its potential as a predictor for these illnesses [20-23]. However, attention must be given to idiopathic thrombocytopenia, as high RDW values can significantly correlate with thrombocytopenia, characterized by lower PLT levels. This is indicative of some coagulopathies and may suggest that in illnesses where PLT levels decrease and RDW values increase, the RDW/PLT ratio will be elevated [24]. Typically, increased RDW values are associated with anemia due to iron deficiency or insufficient vitamin B12 intake, particularly when accompanied by altered oxygen metabolism, such as in heart failure, which impacts ICU mortality and morbidity [25].

Limitations

Several limitations of our study should be noted. Firstly, as a retrospective observational study, it is subject to potential selection and confounding biases. Secondly, variations in individual pain tolerance may lead to inconsistent blood sample collection times. Finally, while the data were obtained from our hospital database and are presumed to be representative of the general population, we could not compare the RDW/PLT ratio with the BISAP score due to the absence of prior research on this specific comparison.

## Conclusions

New biochemical-derived ratios hold significant potential for predicting the severity of AP concerning morbidity and mortality. Our results demonstrate a significant difference in RDW/PLT ratios between patient severity groups classified by BISAP scores (values less than 3 versus 3 or greater). We propose that the RDW/PLT ratio could be a valuable predictor for assessing AP severity. However, further research is needed to fully explore and validate the utility of the RDW/PLT ratio in AP patients.

## References

[1] Karabuga B, Gemcioglu E, Konca Karabuga E, Baser S, Ersoy O (2022). Comparison of the predictive values of CRP, CRP/albumin, RDW, neutrophil/lymphocyte, and platelet/lymphocyte levels in determining the severity of acute pancreatitis in patients with acute pancreatitis according to the BISAP score. Bratisl Lek Listy.

[2] Bedel C, Türkoğlu S (2020). Red cell distribution width and red cell distribution width to total serum calcium ratio as predictors of mortality in acute pancreatitis: a retrospective cohort study. Eur Res J.

[3] Gliem N, Ammer-Herrmenau C, Ellenrieder V, Neesse A (2021). Management of severe acute pancreatitis: an update. Digestion.

[4] Walkowska J, Zielinska N, Tubbs RS, Podgórski M, Dłubek-Ruxer J, Olewnik Ł (2022). Diagnosis and treatment of acute pancreatitis. Diagnostics (Basel).

[5] Mathuram Thiyagarajan U, Ponnuswamy A, Thomas R (2021). Can inflammatory markers foretell aetiology and prolonged hospitalisation in acute pancreatitis?. Cureus.

[6] Mederos MA, Reber HA, Girgis MD (2021). Acute pancreatitis: a review. JAMA.

[7] Banks PA, Bollen TL, Dervenis C (2013). Classification of acute pancreatitis—2012: revision of the Atlanta classification and definitions by international consensus. Gut.

[8] Gao W, Yang HX, Ma CE (2015). The value of BISAP score for predicting mortality and severity in acute pancreatitis: a systematic review and meta-analysis. PLoS ONE.

[9] Li CL, Jiang M, Pan CQ, Li J, Xu LG (2021). The global, regional, and national burden of acute pancreatitis in 204 countries and territories, 1990-2019. BMC Gastroenterol.

[10] Sekimoto M, Takada T, Kawarada Y (2006). JPN Guidelines for the management of acute pancreatitis: epidemiology, etiology, natural history, and outcome predictors in acute pancreatitis. J Hepatobiliary Pancreat Surg.

[11] Rezan TK, Firdevs T, Zeynep K, Umut P, Cemil K, Esad TF (2019). Role of redcell distribution weight in predicting disease severity, mortality and complication in patients with acute pancreatitis. Signa Vitae.

[12] Kılıç MÖ, Çelik C, Yüksel C, Yıldız BD, Tez M (2017). Correlation between Ranson score and red cell distribution width in acute pancreatitis. Ulus Travma Acil Cerrahi Derg.

[13] Cheng T, Liu BF, Han TY, Pan P, Liu JZ, Yu H (2021). Efficiency of red cell distribution width in predicting severity and mortality of patients with acute pancreatitis: a protocol for systematic review and meta-analysis. Medicine (Baltimore).

[14] Ljuca A, Rizvanović N, Ljuca S, Jahić A (2023). Red blood cell distribution width as a predictor of outcome in intensive care unit: a retrospective cohort study. Med Glas (Zenica).

[15] Kim KM, Nerlekar R, Tranah GJ, Browner WS, Cummings SR (2022). Higher red cell distribution width and poorer hospitalization-related outcomes in elderly patients. J Am Geriatr Soc.

[16] Cetinkaya E, Senol K, Saylam B, Tez M (2014). Red cell distribution width to platelet ratio: new and promising prognostic marker in acute pancreatitis. World J Gastroenterol.

[17] Chen C, Xia SH, Chen H, Li XH (2008). Therapy for acute pancreatitis with platelet-activating factor receptor antagonists. World J Gastroenterol.

[18] Ruβwurm S, Dohrn B, Oberhoffer M, Meier-Hellmann A, Krause S, Lösche W, Reinhart K (2000). Platelets and platelet-activating factor acetylhydrolase in septic patients. Crit Care.

[19] Tsukioka K, Matsuzaki M, Nakamata M, Kayahara H, Nakagawa T (1996). Increased plasma level of platelet-activating factor (PAF) and decreased serum PAF acetylhydrolase (PAFAH) activity in adults with bronchial asthma. J Investig Allergol Clin Immunol.

[20] Song CS, Park DI, Yoon MY (2012). Association between red cell distribution width and disease activity in patients with inflammatory bowel disease. Dig Dis Sci.

[21] Lou Y, Wang M, Mao W (2012). Clinical usefulness of measuring red blood cell distribution width in patients with hepatitis B. PLoS ONE.

[22] Qiu L, Chen C, Li SJ (2017). Prognostic values of red blood cell distribution width, platelet count, and red cell distribution width-to-platelet ratio for severe burn injury. Sci Rep.

[23] Mahmood NA, Mathew J, Kang B, DeBari VA, Khan MA (2014). Broadening of the red blood cell distribution width is associated with increased severity of illness in patients with sepsis. Int J Crit Illn Inj Sci.

[24] Nagajothi N, Braverman A (2007). Elevated red cell distribution width in the diagnosis of thrombotic thrombocytopenic purpura in patients presenting with anemia and thrombocytopenia. South Med J.

[25] Xanthopoulos A, Giamouzis G, Dimos A, Skoularigki E, Starling RC, Skoularigis J, Triposkiadis F (2022). Red blood cell distribution width in heart failure: pathophysiology, prognostic role, controversies and dilemmas. J Clin Med.

